# Capability of a neck worn device to measure sleep/wake, airway position, and differentiate benign snoring from obstructive sleep apnea

**DOI:** 10.1007/s10877-014-9569-3

**Published:** 2014-03-06

**Authors:** Daniel J. Levendowski, Bratislav Veljkovic, Sean Seagraves, Philip R. Westbrook

**Affiliations:** 1 Advanced Brain Monitoring Inc., Carlsbad, CA USA; 2 Professor Emeritus, School of Medicine, University of California in Los Angeles (UCLA), Los Angeles, CA USA

**Keywords:** OSA, Positional, Snoring, Screening, Actigraphy, Validation

## Abstract

To evaluate the accuracy of a neck-worn device in measuring sleep/wake, detecting supine airway position, and using loud snoring to screen for obstructive sleep apnea. Study A included 20 subjects who wore the neck-device during polysomnography (PSG), with 31 records obtained from diagnostic and split-night studies. Study B included 24 community-based snorers studied in-home for up to three-nights with obstructive sleep apnea (OSA) severity measured with a validated Level III recorder. The accuracy of neck actigraphy-based sleep/wake was measured by assessing sleep efficiency (SE). Differences in sleep position measured at the chest and neck during PSG were compared to video-editing. Loud snoring acquired with an acoustic microphone was compared to the apnea-hypopnea index (AHI) by- and acrosspositions. Over-reported SE by neck actigraphy was inversely related to OSA severity. Measurement of neck and chest supine position were highly correlated with video-edits (r = 0.93, 0.78). Chest was bias toward over-estimating supine time while the majority of neck-device supine position errors occurred during CPAP titrations. Snoring was highly correlated with the overall, supine, and non-supine PSG-AHI (r = 0.79, 0.74, 0.83) and was both sensitive and specific in detecting overall, supine, and non-supine PSGAHI >10 (sensitivity = 81, 88, 82 %; specificity = 87, 79, 100 %). At home sleep testing-AHI > 10, the sensitivity and specificity of loud snoring was superior when users were predominantly non-supine as compared to baseline (sensitivity = 100, 92 %; specificity = 88, 77 %). Neck actigraphy appears capable of estimating sleep/wake. The accuracy of supine airway detection with the neck-device warrants further investigation. Measurement of loud snoring appears to provide a screening tool for differentiating positional apneic and benign snorers.

## Introduction

It has been estimated that the worldwide prevalence of snoring ranges from 30 to 50 % of the adult population [1–9]. The odds of being a habitual snorer are greater in men [1, 3, 8, 9], with the prevalence further increased by age [1, 3, 7, 10], weight [1, 5, 6], alcohol consumption [1, 5], and/or when pregnant [11–14]. Snoring is an independent risk factor for obstructive sleep apnea (OSA) [15, 16]. Approximately 50 % of those who snore are likely to have OSA [17–20], and a substantial majority is undiagnosed [20]. The vibrations caused by heavy snoring have also been suggested as an independent risk factor for carotid atherosclerosis [21–24]. Of the 15–25 % of the adult population who are benign snorers [i.e., apnea-hypopnea index (AHI) < 5], most snore predominantly in the supine position [25, 26].

Recognition of the clinical importance of OSA risk factors and treating OSA is increasing worldwide, however an inexpensive, objective tool to differentiate those in need of a diagnostic sleep study from those with just benign snoring remains elusive. Such a tool might also be useful for large epidemiological assessments of snoring and OSA prevalence, the impact of snoring on cardiovascular disease, or the changes in snoring during pregnancy.

Previous reports suggest that the measurement of snoring with an acoustic microphone holds potential for non-invasively estimating sleep disordered breathing severity [27–31]. Fiz et al. [28] found that as AHI severity increases, the acoustics change, suggesting the maximum snoring frequency is reduced, the mean snoring intensities increase, and the ratio between the 100 and 500 Hz power expand. With respect to identifying excess tissue associated with airway collapse, fast-Fourier transform of high frequency snoring showed that palatal snoring lies in the lower frequencies of the sound spectrum with snoring frequencies increase stepwise from pharyngeal lateral wall, tongue based, and epiglottis obstruction [32]. Mikami et al. [30] showed that the fundamental frequency and maximum amplitudes in frequency spectrum can be used to distinguish oral snoring sounds in patients with and without sleep apnea. Fiz et al. [28] reported that receiver operating curves using acoustic snoring could provide sensitivities and specificities of 98.0 and 71.4 for an AHI ≥ 5 and 80.0 and 90.0 for AHI ≥ 15 so long as sleeping position was factored into the snoring parameters. Abeyratne et al. [33] validated a OSA screening tool that used a multi-feature class analysis of snoring sounds acquired during laboratory polysomnography that provided sensitivities and specificities of 93 % with an AHI cut-off of 15 events/h. Ben-Israel et al. [27] achieved a sensitivity of 0.87 and a specificity 0.80 with a non-contact microphone placed 1 meter above the bed at a sleep center.

Measuring the influence of position on snoring and/or OSA is important given 65 % of benign snorers are positional and over 70 % of patients with mild to moderate OSA are twice as severe supine as compared to non-supine [25]. Assessing snoring patterns relative to position may prove useful in monitoring outcomes with position restriction treatment, as well as other OSA therapies.

This report provides a description of a neck-worn device and a retrospective assessment of its capability to accurately measure sleep position and sleep/wake, and differentiate benign snorers from those with clinically relevant OSA.

## Methods

### Description of neck-device

**Fig. 1:**
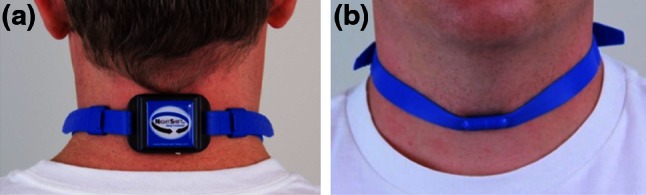
Photograph of neck-device from **a** back and **b** front

The battery-powered, neck-worn device (Night Shift™, Advanced Brain Monitoring, Carlsbad, CA, USA) weighs 44 g and includes electronics housed in a 5.5 (l) × 3.8 (w) × 1.6 (h) cm enclosure affixed on the back of the neck with an adjustable non-latex silicone rubber strap secured by a magnetic clasp (see Fig. 1).

The neck-device measures snoring with a built-in acoustic microphone (PUI audio POM-2246P). The raw audio input is sampled at 2 kHz, and root mean square (RMS) of the digitized signal (12-bit A/D) is calculated using a 100 ms window. The resulting 10 Hz RMS signal is additionally filtered with a 0.5 Hz LP Butterworth 4th order filter. A snore algorithm quantifies each snore based on shape (attack, plateau and decline) and the peak amplitude, prior to conversion to dB. Decibel calibration was performed using a sound level meter and recorded snoring sounds at 12 cm. Snores >40 and 50 dB are tallied across each 30-s epoch. Loud snoring is defined as at least one snore with a magnitude ≥50 dB.

The percentage of time snoring >50 dB is then determined for overall, supine, and non-supine epochs characterized as sleep by actigraphy. Snoring indicative of clinically relevant sleep disordered breathing is based on >50 dB snoring in ≥10 % of neck-based sleep time.

A three-axis accelerometer is used to measure neck position and actigraphic sleep versus wake. Neck positions are reported as upright, supine, lateral left, lateral right, and prone. Upright is assigned when the neck angle is ≥60°. Supine is assigned when the neck angle to the left/right is <43°. Lateral left or right was assigned when the neck angle exceeded 47°. The device remained in the previously assigned position when the neck angle fell between 43° and 47°. Prone is the mirrored position of supine with the additional requirement that the Z axis is <−15°. If the device is worn upside down, the supine position will be accurately measured however lateral left and right will be inversed.

Sleep versus wake is measured in 30-s epochs using a threshold applied to the median filter output derived from the three X, Y, and Z signals. If any of the three signals have an angle <50° and exceeds the actigraphy threshold, the epoch is classified awake. Periods with gross movement extend the wake classification for up to 3 epochs. The initial 10-min after the device is turned on is automatically classified as wake.

Two × 1G haptic motors optionally provide vibro-tactile feedback when the supine position is detected. Positional feedback, initiated at a very low frequency/duration, is gradually increased until the user exits the supine position. When used for positional therapy, vibro-tactile feedback is defaulted to initiate 15-min after the device is turned on, to allow the user time to fall asleep. The user can optionally delay feedback for 30-min or set the device for immediate feedback. The device can also be used only as a recorder with positional feedback disabled.

Data are acquired and analyzed in real time with derived measures for sleep/wake, snoring magnitude, and position for each 30-s epoch saved to the microcontroller flash memory. The memory can store up to six nights’ of detailed snoring, sleep, and position measures (one set of values for each 30-s epoch), summary of key daily parameters by month for 4 months, and the average values across the days in the month for 12 months. This information is accessed via assessment/compliance reports generated in HTML format from a web-enabled portal. The neck-device can record and provide vibro-tactile feedback for three nights before charging is required.

For this study, prototype devices were used that allow raw data to be saved to a memory card for off-line analysis using software which allows the records to be synchronized.

### Study A

Consecutive volunteers scheduled for laboratory polysomnography (Complete Sleep Solutions, Murrieta, CA, USA) between the ages of 18 and 75 with a body mass index (BMI) <35 were recruited to wear the neck-device. Twenty subjects were consented under a protocol approved by the Chesapeake Institutional Review Board (IRB). This cohort included fifteen males and five females with a mean age of 46 ± 13.2 years and a mean BMI of 29 ± 4.1 kg/M^2^.

For this study, the neck-device was set to record mode (i.e., no positional feedback). The device was applied and turned on by the technician. Patients were instructed to sit upright in bed for 1 min just prior to lights out and just after lights on, to provide a means to synchronize the polysomnography (PSG) and the neck-device records.

The sleep studies were conducted and scored according to the American Academy of Sleep Medicine (AASM) criteria with 3 % hypopnea desaturation [34]. Alice three or five systems were used with Pro-Tech nasal pressure transducers and Pro-Tech respiratory induced plethysmography effort sensors (Philips Respironics, Monroeville, PA, USA), and either Pro-Tech or SleepMate (Ambu, Inc. Glen Burke, MD, USA) actigraphy-based body (chest) position sensors.

### Study B

Twenty-five individuals responding to a newspaper advertisement met the inclusion criteria of: (a) age between 30 and 85, (b) slept nightly with a bed partner who complained about their snoring, and (c) were not currently being treated with CPAP for OSA. Subjects were consented with a protocol approved by the BioMed IRB. This cohort included 14 males and 10 females with a mean age of 44 ± 10.5 years and a mean BMI of 31 ± 7.7 kg/M^2^ (range 22–57). Eighty-four percent were identified as being at high OSA risk by the STOP questionnaire [35] or by the ARES questionnaire [36, 37], with conflicting results in two cases. A prior diagnosis of OSA, high blood pressure and/or depression was reported in 12, 12 and 23 % of the cases, respectively.

Subjects were instructed to wear the neck-device concurrent with a multi-channel home sleep testing (HST) device (ARES™, SleepMed, Boca Raton, FL, USA) for three nights. Night 1 was intended to provide a baseline, so the neck-device was set to record mode. On nights 2 and 3, the neck-device was set to feedback mode using preliminary supine detection parameters which under-reported supine sleep (e.g., neck angle < 35° vs. < 43°).

The HST was affixed to the forehead and measured airflow from a cannula and pressure transducer, pulse and oximetry using reflective plethysmography, quantified snoring (dB) with a calibrated acoustic microphone, and head movement and head position by actigraphy. Automated scoring algorithms were applied off-line to compute the AHI. Apneas, based on a 10-sec cessation of airflow detected by the automated algorithms, were included in the apnea-index (AI) and the AHI. Hypopnea events required a 50 % reduction and recovery in airflow, and a minimum 3.5 % reduction in SpO_2_ and at least a 1.0 % recovery. Sleep time was determined behaviorally, based on actigraphy, snoring, and airflow patterns. The algorithms and accuracy of the automated scoring used to calculate the AHI have been previously reported [38–40].

Just prior to lights out, subjects were instructed to turn both devices on and perform a slow, loud 10-count, captured by both acoustic microphones and used to synchronize the records. Each morning subjects completed brief surveys to assess their sleep quality.

### Data analysis

#### Study A

The Alice software was used to export: (a) EDF files to obtain epoch level resolution of chest position, (b) sleep stages for each epoch, (c) recording and sleep time, and (d) apnea/hypopnea indexes by, and across, positions. Technician notes from the video review were used to edit the body position.

To compare PSG and neck-device epochs classifications of sleep or wake and supine or non-supine, the clock times from the files were aligned, with accurate synchronization confirmed by the change to the upright position at the start of the recording. Comparisons of sleep versus wake were measured from lights out until lights on. Recording time was used to compare percentage of supine time from the chest and neck. Video recordings were reviewed to provide the gold-standard for classification of the supine position for both the chest and neck.

The overall percent time snoring ≥50 dB from the neck (loud snoring) was computed during periods detected as sleep by the neck actigraphy. From these sleep epochs, the supine and non-supine percent times snoring ≥50 dB were derived. Reporting of the percentage of positional time snoring required a minimum of 12-min of neck actigraphy-based sleep time.

For the 11 participants who underwent a split-night study, snoring and AHI values were derived separately for the diagnostic and the CPAP titration periods. “Appendix 1” provides line data for the 20 subjects and 31 records used for analyses.

#### Study B

Prior to off-line analysis, both the HST and neck-device files were opened to identify the segment of the ARES EDF file which needed to be excluded so that the 10-counts were aligned.

### Statistical analyses

Pearson correlations and Bland–Altman plots were used to characterize the association and differences between the paired measures. Two-tailed *t* tests, which assumed equal variances, were used to measure significant between-measure differences. Cross-tabulations were used to compute the sensitivity, specificity, positive predictive value (PPV) and negative predictive value (NPV) between the overall, supine, and non-supine AHI and when loud snoring (i.e., ≥50 dB) exceeded 10 % of sleep time. Repeated measures analysis of variance (ANOVA) was used to assess significant changes in percent time supine across nights. Subjects identified with positional obstructive sleep apnea (POSA) required an overall AHI ≥ 5 and a supine/non-supine AHI ratio ≥ 2.0 (based on >12-min of positional sleep time).

## Results

### Study A

**Fig. 2:**
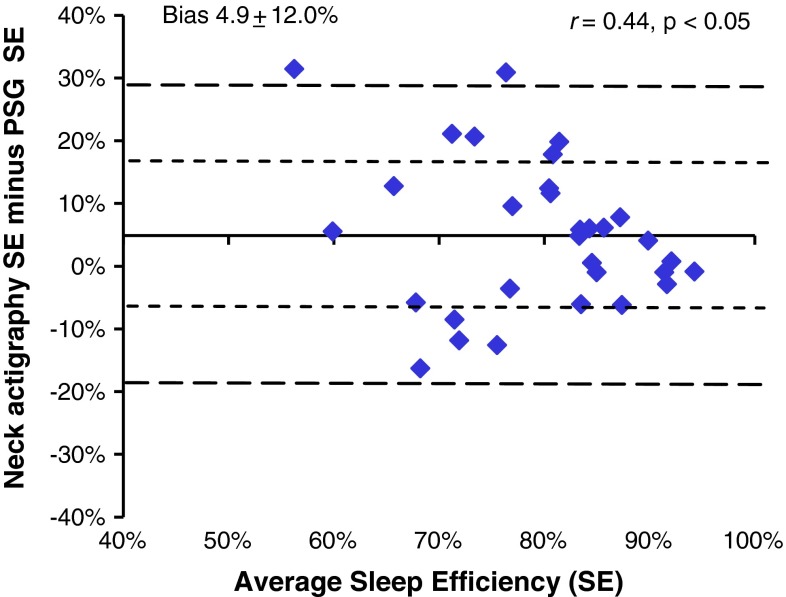
Bland–Altman plot comparing sleep efficiency between PSG and neck actigraphy

The mean (±SD) sleep efficiency (SE) based on electrophysiology and actigraphy were 76.8 ± 12.3 and 81.8 ± 10.0 %, respectively. No significant differences in SE were observed between PSG and the neck-actigraphy during the diagnostic studies (73.8 ± 12.1 and 78.4 ± 10.9, respectively) or during the CPAP trials (82.4 ± 11.1 and 87.9 ± 3.7 %). Moderate agreement was observed between the PSG and neck actigraphy SE (*r* = 0.44). Neck actigraphy bias toward over-reporting sleep time (Fig. 2.) decreased as OSA severity increased. Biases of 9.9 % observed for those with no OSA (AHI < 5), 5.6 % for those with mild to moderate OSA (AHI 5–29) and −4.9 for those with severe OSA (AHI ≥ 30).

**Fig. 3:**
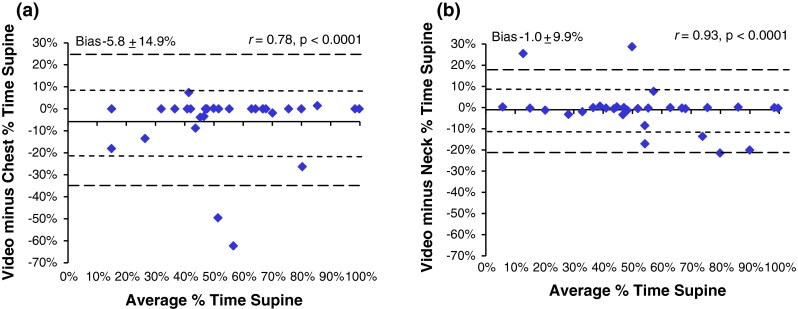
Bland–Altman plots between edited and unedited actigraphy-based position from lights out to lights on from **a** chest and **b** neck

**Fig. 4:**
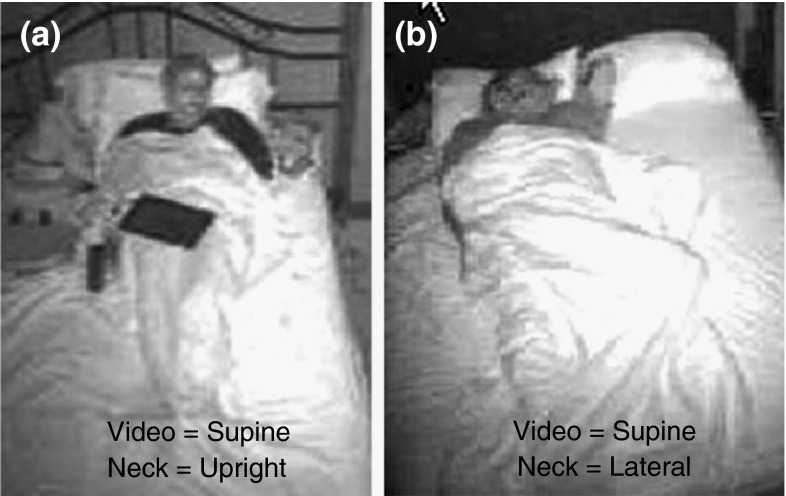
Photos of two cases of gross under-reporting of supine by the neck-device with **a** S22 misclassified as upright by neck and supine by video, and **b** S12 misclassified as lateral by neck

Very strong, significant agreements were observed between the video-edited supine position and those measured by chest and neck (*r* = 0.78 and 0.93 respectively) (Fig. 3). When Subject 12 was removed, the correlation between chest and video-edited chest increased from 0.78 to 0.88. The chest transducer over-reported supine time by >5 % in six studies, with an average error of 80 min (range 16–284). The average error in supine time decreased to 39 min when subject 12 was removed. In six cases, the absolute difference between supine time by neck position and video exceeded 5 %. In four cases, supine sleep time was over-reported by neck position by an average of 28 min (range 16–60 min), and in two cases, neck position under-reported supine sleep time by 18 and 38 min. In only two cases, the neck and chest were reported in conflicting positions by video editing (Fig. 4). Subject 18 spent an additional 18 min supine based on the position of the neck; subject 20 spent 15 additional minutes supine based on chest position.

During the diagnostic studies, 30 % of the subjects had an AHI < 5. Significant agreements (*p* < 0.0001) were observed between the percentages of sleep time snoring ≥50 dB from the neck and the overall, supine, and non-supine AHI (*r* = 0.79, 0.74, and 0.83, respectively).

For an overall AHI ≥ 10, the sensitivity and specificity of loud snoring (i.e., 50 dB for ≥10 % of sleep time) were 81 and 87 %, respectively, with a PPV and NPV of 87 and 81 % respectively. The sensitivity and NPV improved to 87 and 88 %, respectively, with a clinical cut-off of AHI ≥ 15. At an AHI ≥ 5, the absence of loud snoring effectively ruled out mild OSA in 92 % of the cases, and the presence of loud snoring provided a 93 % probability of correctly identifying those with OSA.

Loud snoring was more robust in identifying important sleep disordered breathing in the non-supine position as compared to the supine position. For a non-supine AHI ≥ 10, the sensitivity and specificity were 82 and 100 %, and the PPV and NPV were 100 and 90 %, respectively. At an AHI ≥ 15, the non-supine sensitivity and specificity were 100 and 95 %. In the supine position, the clinical cut-off AHI ≥ 10 provided the optimal accuracy with a sensitivity, specificity, PPV, and NPV of 88, 79, 82, and 85 %, respectively (Table 1).

**Table 1:** Accuracy of loud snoring (>10 % of neck-based sleep time above 50 dB) in detecting PSG-AHI ≥ 5, 10, and 15

	Overall PSG-AHI			Supine PSG-AHI			Non-supine PSG-AHI		
	>5 (%)	≥10 (%)	≥15 (%)	>5 (%)	≥10 (%)	≥15 (%)	>5 (%)	≥10 (%)	≥15 (%)
Sensitivity	77.8	81.3	86.7	78.9	87.5	86.7	75.0	81.8	100.0
Specificity	92.3	86.7	87.5	81.8	78.6	73.3	100.0	100.0	95.0
PPV	93.3	86.7	86.7	88.2	82.4	76.5	100.0	100.0	88.9
NPV	75.0	81.3	87.5	69.2	84.6	84.6	84.2	89.5	100.0

### Study B

Of the 24 subjects who successfully completed night 1, 21 also completed night 2 and 19 completed night 3. All available data were analyzed with the exception of one subject excluded due to missing data on Night 1 baseline (forgot to turn the device on) which limited comparisons to feedback nights 2 and 3. Subjects 9 and 13 had missing data on nights 2 and/or 3 due to not turning the neck-device on. Subjects 19 and 21 turned the neck-device off on nights 2 and 3 due to complaints of repetitive buzzing. Subject 10 turned the HST off during night 3. “Appendix 2” provides line data used for the analyses.

**Fig. 5:**
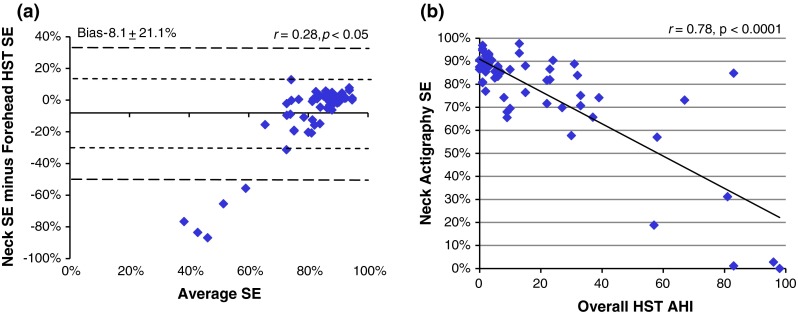
**a** Bland–Altman plot between sleep efficiency (SE) measured by actigraphy from the neck and forehead HST across all nights, and **b** correlation plots between HST-AHI and sleep efficiency (SE) by neck actigraphy across all nights

From this community-based cohort, 33 % were benign snorers, 42 % had mild or moderate OSA, and 25 % had severe undiagnosed OSA (i.e., overall AHI ≥ 30). Significant differences were observed in the sleep time and SE measured by HST and neck-actigraphy (*p* < 0.05) (Fig. 5a). Figure 5b confirms that the reduction in SE accuracy, measured by neck actigraphy, was attributed to very severe OSA and supported the addition of a SE threshold to further characterize OSA with the neck device.

As was observed during PSG, a very strong agreement was noted between those with loud in-home snoring plus a SE ≥ 25 and HST-AHI (*r* = 0.76). When the combination of SE and loud snoring thresholds (i.e., SE ≤ 25 or snoring ≥10 % of sleep time) were compared to HST-AHI severity, the accuracies were similar to those observed during PSG. Specificities were slightly inferior at baseline, and superior on nights 2 and 3 when subjects spent the majority of time in the non-supine position. For an overall AHI ≥ 10 at baseline, the sensitivity and specificity of loud snoring were 92 and 77 %, respectively, with a PPV and NPV of 79 and 91 % respectively. For an AHI ≥ 15, the sensitivity and NPV improved to 100 and 100 %, respectively, while the specificity and PPV decreased slightly to 73 and 71 %, respectively. On nights 2 and 3, the sensitivity, specificity, PPV and NPV for an AHI ≥ 10 were 100, 88, 84, and 100 %, respectively (Table 2).

**Table 2:** Accuracy of loud snoring and SE in detecting HST-AHI ≥ 5, 10, and 15

	Night 1: HST-AHI at baseline			Nights 2 and 3: HST-AHI w/positional feedback		
	>5 (%)	≥10 (%)	≥15 (%)	>5 (%)	≥10 (%)	≥15 (%)
Sensitivity	70.6	91.7	100.0	85.0	100.0	100.0
Specificity	75.0	76.9	73.3	90.0	87.5	80.8
PPV	85.7	78.6	71.4	89.5	84.2	73.7
NPV	54.5	90.9	100.0	85.7	100.0	100.0

Sixty-nine percent of subjects with an AHI ≥ 5 had POSA at baseline based on forehead position. The association between the percentage of sleep time supine reported from the neck and forehead was moderate (*r* = 0.38) with substantial variability (mean of differences 3 ± 26.7 %). Feedback significantly reduced the percentage of time supine on nights 2 and 3 based on neck position (*F* = 13.75, *p* < 0.0001) but not by forehead position.

## Discussion

The purpose of this study was to evaluate the capabilities of a neck-device which uses actigraphy to measure sleep/wake and sleep position, and an acoustic microphone to record loud snoring. Studies conducted during PSG provide the gold-standard comparisons for the initial validation. Inclusion of studies during CPAP titration provides a second condition for assessment of sleep/wake, position, and snoring in over half the cases. Results were then cross-validated in-home with a community-based cohort of snorers across multiple nights with and without supine avoidance feedback.

To our knowledge, this is the first report on the use of neck actigraphy to detect sleep/wake. Typically, actigraphy accuracy is challenged by the over-estimation of sleep due to long periods of inactivity. In OSA patients, actigraphy accuracy is challenged by correct identification of sleep fragmentation and movement resulting from sleep-disordered breathing. For the 31 comparisons, absolute SE errors exceeded 10 in 39 % of the records. By way of comparison, wrist-actigraphy based Actiwatch-64 and Fitbit reported measurement errors of 50 and 58 %, respectively, in 24 healthy subjects [41]. For those with an AHI < 5, the neck-device was equivalent in accuracy to Actiwatch and superior to Fitbit in the over-reporting of sleep (i.e., bias: 9.9 vs. 9.3 and 14.5 % respectively). The strong association between over-reporting of wake neck actigraphy and severe OSA was more apparent in the home studies. To achieve reasonable sleep/wake accuracy without the benefit of off-line processing of multiple parameters (i.e., actigraphy, snoring, and airflow), the wake threshold for the neck-device was set to twice the actigraphy threshold selected for forehead HST. One limitation of this study was that many conventional measures, such as sleep time, sleep latency, and wake after sleep onset, were not reported due to the number of split-night studies. A more rigorous validation of neck-actigraphy conducted during PSG studies with sleep times exceeding 5 h will be reported elsewhere.

A second objective of this study was to compare sleeping positions derived from transducers placed in different locations (e.g., chest, neck and head). During PSG, a substantially greater number of gross supine position detection errors were observed for both chest and neck as compared to Bignold [42], with both methods tending to over-report the supine position. Measurement of supine position during CPAP titration provides one explanation for the discrepancy. Four of six gross supine position errors by neck position, and two of six gross errors by chest position, occurred while on CPAP when patients tend to direct their head/neck toward the unit and then settle into the same position for long periods of time. Sleeping in a semi-upright position confounded both the chest and neck transducers, with chest over-reporting the supine position by 284 min and neck under-reporting the supine position by 116 min. Considering the impact of position detection errors within the construct of a feedback device, false-positive supine detection may be interpreted as a nuisance and could contribute to non-compliance. False-negative detection would potentially result in non-efficacious treatment. Investigation of the false negative neck device errors during CPAP titration showed the neck was in the overlap range between supine and lateral with the head and neck rotated by more than 30° and the airway in a position less susceptible to collapse [43, 44]. A prospective cross-validation of the accuracy of neck-based position assessment will be reported separately.

A third objective of this study was to assess whether loud snoring could be effective in screening for important sleep-disordered breathing. We conducted this evaluation under laboratory and home-based conditions, included at least 30 % of participants without sleep disordered breathing, recruited from the community, and assessed patients under treatment conditions (i.e., CPAP titration and position restriction). In previous investigations, the percentage of time snoring based on the magnitude and duration of each snore >40 dB, but not >50 dB, was a significant predictor of positional OSA and successful outcomes with oral appliance therapy [15, 37, 44, 45]. Because snoring modulates across hypopneas and terminates during apneas, cases were reported in which the percent time snoring increased with oral appliance therapy when apneas were converted to hypopneas. We hypothesize that the strong association between snoring and AHI across a wide range of OSA severities, during both PSG and HST, resulted from a new approach whereby entire 30-s epochs were assigned loud snoring when at least one snore exceeded 50 dB in the duration. Including only epochs classified as sleep may also have assisted in improving snoring sensitivity and specificity.

Loud snoring for more than 10 % of the night was effective in identifying important sleep disordered breathing across conventional OSA clinical cut-offs and was more accurate when subjects were in the non-supine position. During non-supine sleep, the optimal sensitivity was obtained with PSG-AHI ≥ 15 (100 %) and optimal specificity obtained with PSG-AHI ≥ 5 % (100 %). During supine sleep, a cut-off of PSG-AHI ≥ 10 provided the highest sensitivity (88 %), with PSG-AHI ≥ 5 providing the best specificity (82 %). When a HST-AHI ≥ 10 was applied to the baseline sessions, the sensitivity and specificity from loud snoring were 91 and 77 %, respectively. When patients were predominantly non-supine (i.e., nights 2 and 3), the accuracy improved to 100 and 88 %, respectively. The accuracy in screening for OSA with the neck-device was similar to approaches which utilized a non-contact acoustic microphone [27, 33]. Unlike the non-contact snoring approaches, the neck-device can estimate positional severity.

These findings suggest that the neck-device is capable of identifying important sleep disordered breathing in most cases. When loud snoring is measured with an acoustic microphone in combination with actigraphy-based sleep time from the neck, the SE must be considered in those with very severe OSA. The addition of SE did not affect the results obtained during PSG data because all SE were above the minimum threshold. This finding may be attributed to short-duration diagnostic sleep times resulting from the obvious need to initiate CPAP therapy.

The improved AHI detection accuracy of loud snoring in the non-supine position, during both PSG and HST, suggests possible interaction between the acoustic microphone and bed-coverings. Two false-negative findings did not appear to be solely attributed to the pillow because snoring below the loud snoring threshold was observed in both the supine and non-supine positions. Conversely, a false-positive result from loud snoring did not appear to be due to CPAP mask leakage because CPAP pressure and AHI were both low. In most cases, the vibration sounds above 50 dB seemed to be effective in assessing OSA severity at baseline and under treatment conditions. Additional studies should be conducted to investigate conditions which contribute to false-positive and false-negative results. For example, it’s possible that long duration apnea or hypopnea events contribute to false negative results at AHI ≥ 15. Additional profiling of snoring patterns such as excluding snores which occur during movement, may further improve the detection capability of this measure.

Given the high prevalence of loud snoring worldwide (see “Appendix 3”), and the impact of undiagnosed OSA on both co-morbidities and medical costs, the need exists for easy, inexpensive, and accurate differentiation of benign snorers from those who should undergo a diagnostic study for OSA. As a screening tool, the results obtained with the neck-device were comparable to a single-channel Apnea Link nasal pressure device [45], with both devices capable of being self-applied and worn in the home. The neck-device provided superior sensitivity and specificity, as compared to ApneaLink during PSG, when clinical cut-offs of AHI ≥ 5 and ≥10 were applied and inferior detection accuracy when AHI ≥ 15 was used. One weakness of the neck-device is the potential for cross talk from a snoring bed partner. Bench-tests suggest that loud bed-partner snoring can exceed 40 dB but rarely 50 dB. Unlike the ApneaLink, screening of apneic and non-apneic snorers with the neck-device can include the assessment of airway position by comparing overall snoring time to supine and non-supine snoring time [25, 26].

Previous reports suggest that POSA is prevalent in 53 % of patients with sleep disordered breathing (i.e., AHI ≥ 5 plus supine AHI at least two times greater than the non-supine AHI) [48, 49]. In the Study A group, 70 % had an AHI ≥ 5 while 45 % of the cohort was identified with POSA based on body position. POSA prevalence would increase to 50 % and match previous reports if subjects with supine/non-supine ratios of 1.9 and 1.8 were included. The conventional definition of POSA (i.e., supine/non-supine ratio ≥2.0) has assumed a 4 % desaturation criteria [25, 48–51]. A relaxed definition of POSA may be necessary when a 3 % desaturation criteria is applied. It would be expected that a greater number of non-supine events would meet the AHI criteria given oxyhemoglobin desaturation may be less severe when lung volume is somewhat larger.

The concordance between the neck-and-chest-derived percentage of time supine was much stronger than neck and forehead supine sleep times. The prevalence of POSA detected by forehead position across a range of OSA severities was similar to previous estimates of those with mild to moderate OSA [48, 49]. Excessive measurement of supine sleep may also explain why significant changes in percent time supine from the neck were observed on nights 2 and 3 but not the forehead. As a result of the discrepancies, the agreement between positional HST-AHI and loud snoring were not compared. The accurate detection of a supine airway from the forehead is difficult because the head can be in the same angle with the torso either laterally or supine. The algorithms defining the supine position of the airway from the neck are less complicated due to its more restricted rotation. Others have argued that both head and trunk positions are clinically relevant in assessment of OSA because neither exclusively define the position of the airway [43, 44]. This study suggests that neck position may provide an alternative approach to measuring the impact of position on airway collapsibility.

One limitation of this study was the limited assessment of the benefit of supine avoidance feedback. Study B was designed as a usability study for the neck-device, not as a treatment outcome study. It was not anticipated that a quarter of the community-based snorers would have severe undiagnosed OSA and that finding supports the need for a simple, inexpensive screening tool. Proper assessment of positional feedback outcomes, such as those conducted by Van Maanen et al. [49, 50] requires inclusion criteria limited to only those with POSA. Our study with feedback did provide insight as to conditions most conducive for vibro-tactile position therapy. For example, the two subjects, who presumed the device was not working properly because it buzzed every 20–30 min, slept 100 % of the time on their back at baseline. Those who sleep exclusively supine may require a longer training/adaptation period and/or non-compliance may be greater. A report of neck strap being uncomfortable was from a female with a 54.6 cm neck circumference. It’s unclear whether she wore the strap too tight, but it also suggests that extreme adipose tissue around the neck may influence compliance. Given the neck-device was designed to measure compliance, and can be trialed with limited effort, these concerns may be manageable with improved patient selection.

## Conclusions

This report provides a full description of a neck-worn device and an initial assessment of its capabilities. Results suggest that neck actigraphy is capable of estimating sleep/wake and may be useful in predicting severe OSA. Detection of the supine airway by neck actigraphy was equivalent to the measures obtained from the chest. The supine detection accuracy of the neck-device supports further investigation for use with supine-avoidance therapy. Comparison of loud snoring, acquired with a neck-based acoustic microphone, to the AHIs obtained by PSG and HST suggest sensitivities and specificities equivalent to other simple screening tools. The combination of position assessment and OSA severity holds promise as a screening tool to differentiate positional apneic and benign snorers.

## Appendix 1

See Table 3.

**Table 3:** Detailed data by subject used in Study A

Subj. no.	Study type	Recording time-min	Sleep efficiency (%)		Overall		Supine		Non-supine		Supine (%)			
			PSG (%)	Neck (%)	AHI	Snoring (%)	AHI	Snoring (%)	AHI	Snoring (%)	Chest	Chest/video	Neck	Neck/video
S2*	Diag.	247	78.5	74.9	25.7	66.6	44.1	78.9	16.6	58.5	40.9	40.9	41.1	40.9
S2*	CPAP Titr.	205	80.5	86.3	27.3	36.0	44.5	55.5	0.0	0.8	67.8	67.8	68.3	67.8
S3*	Diag.	305	81.8	69.2	54.3	56.3	95.6	61.9	41.6	53.5	32.0	32.0	33.9	32.0
S3	CPAP Titr.	210	93.1	90.2	2.9	2.6	4.3	2.6	1.6	2.7	47.1	47.1	47.1	47.1
S4	Diag.	499	60.9	91.8	1.9	3.8	2.3	4.0	1.3	1.3	71.0	69.1	90.6	69.1
S5*	Diag.	210	84.3	84.8	12.5	5.0	34.4	13.3	1.0	0.0	48.1	39.3	38.6	39.3
S5	CPAP Titr.	228	80.9	85.7	0.7	2.3	0.5	2.5	0.8	1.3	93.4	67.1	80.7	67.1
S6	Diag.	239	75.7	67.2	56.7	51.2	56.5	52.1	57.1	48.9	66.7	66.7	66.9	66.7
S6	CPAP Titr.	194	90.5	84.3	1.7	2.1	1.8	1.2	1.5	5.6	75.6	75.6	75.6	75.6
S11	Diag.	406	40.5	72.0	0.0	10.1	0.0	15.0	0.0	5.4	37.7	45.1	48.4	45.1
S12	Diag.	481	94.7	93.8	1.1	0.0	0.4	0.0	0.0	0.0	87.8	25.5	0.0	25.5
S13	Diag.	146	76.4	60.1	104.9	51.1	106.9	39.5	56.2	60.0	36.6	36.6	36.6	36.6
S13	CPAP Titr.	216	91.9	90.9	20.9	28.7	20.9	28.5	N/A	N/A	99.8	99.8	99.8	99.5
S14*	Diag.	211	57.1	62.6	26.4	24.2	65.5	44.6	11.7	17.5	33.2	19.7	20.9	19.7
S14	CPAP Titr.	187	60.7	81.8	3.2	9.8	0.0	11.1	3.2	9.7	15.0	15.0	15.2	15.0
S15	Diag.	455	72.2	81.8	5.1	1.2	4.8	1.4	6.9	0.0	84.7	86.2	85.9	86.2
S16	Diag.	248	77.8	66.0	60.6	55.8	86.3	66.0	48.1	47.2	49.8	50.0	58.5	50.0
S16*	CPAP Titr.	227	82.6	88.7	23.9	13.5	28.9	13.5	4.7	N/A	80.0	80.0	100.0	80.0
S17	Diag.	409	71.5	91.3	3.5	4.7	6.5	8.5	0.8	1.3	48.3	44.9	44.5	44.9
S18	Diag.	433	81.3	87.3	2.0	0.3	3.0	0.2	1.6	0.3	42.0	42.0	62.8	45.7
S19	Diag.	169	70.7	64.9	80.3	62.7	N/A	N/A	80.3	62.7	24.0	5.9	5.6	5.9
S19	CPAP Titr.	218	87.8	91.9	3.1	4.8	7.7	15.0	1.7	0.4	76.1	26.6	29.8	26.6
S20	Diag.	121	74.8	86.4	58.3	12.4	58.3	12.4	N/A	N/A	98.3	98.3	98.3	98.3
S20	CPAP Titr.	253	91.7	92.5	1.6	2.4	2.4	4.7	0.0	0.0	66.8	66.8	53.4	61.1
S21*	Diag.	348	59.3	72.1	20.6	1.0	32.8	0.9	12.8	1.0	47.3	43.4	44.0	43.4
S22*	Diag.	194	71.9	89.7	80.4	47.6	104.2	53.7	45.0	37.8	62.9	62.9	62.9	62.9
S22	CPAP Titr.	207	63.0	83.7	1.8	2.6	2.7	0.8	0.0	3.7	64.3	64.3	35.5	64.3
S23*	Diag.	435	74.3	86.7	19.0	1.1	25.8	0.8	12.6	1.4	51.6	51.6	52.1	51.6
S24*	Diag.	171	86.5	80.5	59.4	74.6	90.6	51.9	38.5	95.2	47.1	47.1	47.1	47.1
S24*	CPAP Titr.	237	83.3	91.1	5.8	30.2	10.4	53.7	0.0	0.0	55.3	55.3	55.5	55.3
S25	Diag.	403	85.5	84.5	1.6	2.3	5.0	1.5	0.9	3.1	47.6	47.6	48.9	47.6
POSA*	Mean	275	76.8	81.8	24.7	21.5	31.6	23.2	15.4	18.5	58.0	52.3	53.2	52.2
	SD	109	12.3	10.0	29.4	24.2	35.5	25.0	22.7	27.1	21.9	22.8	26.1	22.6

* Positional obstructive sleep apnea

## Appendix 2

See Table 4.

**Table 4:** Detailed data by subject used in Study B

Subj	BMI	Baseline Night 1						Night 2						Night 3					
		AHI	Snoring >50 dB	SE	SE	Sup. time	Sup. time	AHI	Snoring >50 dB	SE	SE	Sup. time	Sup. time	AHI	Snoring >50 dB	SE	SE	Sup. time	Sup. time
			Neck (%)	Neck (%)	Fore-head (%)	Neck (%)	Fore-head (%)		Neck (%)	Neck (%)	Fore-head (%)	Neck (%)	Fore-head (%)		Neck (%)	Neck (%)	Fore-head (%)	Neck (%)	Fore-head (%)
1*	39.3	32	76.9	83.8	89.0	14.6	15.8	30	84.5	57.7	73.3	1.0	0.1	22	94.0	71.6	73.9	1.6	5.3
2*	22.0	5	3.9	85.6	88.8	37.5	27.5	2	4.0	85.4	82.3	4.8	3.6	1	0.8	80.9	81.8	7.0	3.9
3	25.1	15	71.2	88.0	86.7	0.4	0.0	10	62.1	86.4	84.0	1.3	1.6	13	90.2	93.5	89.1	1.6	0.0
4*	25.5	6	2.9	87.5	88.8	19.3	10.1	3	0.3	87.6	89.1	0.8	0.0	1	0.4	86.6	84.8	0.1	0.0
5*	28.4	23	54.7	82.0	81.4	47.7	44.4	39	61.0	74.2	90.0	0.0	56.9	6	11.4	87.9	86.9	15.3	22.1
8*	27.5	23	92.7	86.6	87.6	15.8	15.1	15	72.7	76.4	91.4	0.8	0.0	22	53.4	81.8	86.3	0.0	35.0
9	21.6	3	4.3	88.8	89.6	6.5	11.3	2	1.9	88.7	83.0	3.9	9.4	–	–	–	–	–	–
10	43.7	81	100.0	31.1	86.9	64.1	24.5	57	99.3	18.8	84.3	4.2	0.1	–	–	–	–	–	–
11	37.4	1	1.0	80.7	67.9	50.7	7.1	2	1.0	88.8	85.0	6.0	3.3	2	4.5	77.0	76.6	48.4	16.7
12	28.1	1	7.7	95.4	94.2	11.4	3.5	1	11.2	94.8	94.7	0.1	0.0	3	7.2	93.2	92.0	0.3	0.0
13	24.7	10	11.7	69.5	78.5	52.4	0.8	–	–	–	–	–	–	–	–	–	–	–	–
14*	30.8	7	1.9	85.2	79.9	39.7	56.4	4	4.2	90.5	90.0	16.9	14.1	6	5.0	83.4	82.6	6.2	22.5
15	32.0	83	83.1	84.8	91.1	74.4	63.4	67	87.8	73.1	84.0	43.8	10.3	58	84.2	57.0	88.4	31.8	44.1
16	36.6	96	25.0	2.8	89.7	80.0	10.1	83	77.8	1.1	84.7	55.6	1.5	98	0.0	0.0	76.7	0.0	12.1
17*	27.1	37	15.2	65.7	84.8	2.0	6.1	33	49.1	70.7	91.5	2.2	0.0	27	43.5	69.8	90.2	5.5	2.0
19*	24.2	13	9.3	97.6	89.9	100.0	40.0	–	–	–	–	–	–	–	–	–	–	–	–
21	56.6	1	23.1	96.9	90.7	100.0	33.0	–	–	–	–	–	–	–	–	–	–	–	–
22	25.1	1	2.8	87.4	83.9	37.6	23.2	0	1.4	87.5	87.4	11.2	0.0	3	0.9	89.5	90.2	13.1	0.0
23	24.8	3	1.2	92.7	91.6	76.7	47.0	3	6.0	91.3	90.2	24.9	37.9	2	2.3	88.8	90.7	3.0	63.2
24	29.5	1	14.1	88.1	89.3	8.0	6.2	0	2.9	86.5	89.6	0.0	0.0	0	2.7	90.5	88.3	7.7	0.0
25*	25.1	9	25.3	65.5	85.0	53.6	51.4	9	6.8	68.0	77.6	16.4	98.5	8	6.2	74.2	74.5	4.0	51.4
27*	30.9	5	7.6	82.8	81.7	1.0	12.3	2	5.7	87.3	85.1	17.9	28.0	3	10.5	90.2	86.7	2.2	4.3
28*	27.9	33	59.9	75.1	87.7	43.8	41.2	31	41.6	88.8	84.2	1.4	19.6	24	17.1	90.4	85.6	1.3	23.9
29	35.0	2	0.4	89.6	91.0	7.1	6.5	1	0.0	89.3	88.4	5.2	0.1	2	0.5	92.3	91.0	10.9	0.0
Mean	30.4	20.5	28.9	78.9	86.5	39.4	23.2	18.8	32.4	75.9	86.2	10.4	13.6	15.8	22.9	78.9	85.1	8.4	16.1
SD	7.8	27.3	33.3	21.3	5.6	31.4	19.3	24.7	36.0	24.0	4.9	15.0	24.6	24.5	32.9	21.4	5.8	12.4	19.6

* Positional obstructive sleep apnea

## Appendix 3

See Table 5.

**Table 5:** Prevalence of adult habitual snorers by country and gender

Country	Sample size	Overall (%)	Males (%)	Females (%)	Author
Brazil	3,136	50.5	–	–	Noal et al. [5]
France	850	–	34.6	–	Teculescu et al. [6]
Hong Kong—students	3,063	25.9	–	–	Hui et al. [10]
Italian	1,146	31.0	–	–	De Benedetto et al. [7]
Nigeria	370	31.6	–	–	Adewole et al. [4]
Poland	1,186	75.0	48.0	27.0	Zielinski et al. [9]
Taiwan	4,011	51.9	60.8	42.5	Chuang et al. [2]
Turkey	1,245	38.4	29.5	8.9	Kara et al. [3]
United States	5,201	52.0	33.0	19.0	Enright et al. [1]
United Kingdom	1,075	41.5	29.0	12.5	Davey [8]
